# Anemia rather than hypertension contributes to cerebral hyperperfusion in young adults undergoing hemodialysis: A phase contrast MRI study

**DOI:** 10.1038/srep22346

**Published:** 2016-02-29

**Authors:** Gang Zheng, Jiqiu Wen, Wenkui Yu, Xue Li, Zhe Zhang, Huijuan Chen, Xiang Kong, Song Luo, Xiaolu Jiang, Ya Liu, Zongjun Zhang, Long Jiang Zhang, Guang Ming Lu

**Affiliations:** 1 Department of Medical Imaging, Jinling Hospital, Medical School of Nanjing University, Nanjing, Jiangsu, China; 2 College of Civil Aviation, Nanjing University of Aeronautics and Astronautics, Nanjing, Jiangsu, 210016, China; 3 National Clinical Research Center of Kidney Diseases, Jinling Hospital, Medical School of Nanjing University, Nanjing, Jiangsu, China; 4 Institute of General Surgery, Jinling Hospital, Medical School of Nanjing University, Nanjing, Jiangsu, China

## Abstract

Cerebral hyperperfusion, anemia and hypertension are common in patients with end-stage renal disease (ESRD). Young ESRD adults might afford a better hemodynamic tolerance; however, their cerebral vascular disorders are often overlooked. This phase-contrast MRI study investigated relationships between cerebral blood flow (CBF), anemia and hypertension in young adults undergoing hemodialysis (HD). Blood flows, velocities, and cross-sectional areas of bilateral internal carotid arteries and vertebral arteries were quantified on phase maps in 33 patients and 27 healthy controls. Cerebral oxygen delivery (COD) and vascular resistance were (CVR) were computed based on CBF, hemoglobin and mean arterial pressure (MAP). We found strong correlations among hemoglobin, MAP and CBF. Hemoglobin rather than MAP was directly related to CBF. COD was negatively related to MAP, while CVR was positively related to hemoglobin. The cross-sectional areas of arteries were increased which were directly associated with hemoglobin rather than MAP. HD patients were of elevated CBF, decreased COD and unchanged CVR. Although elevated CBF compensated anemia-induced hypoxia, COD of these patients was still lower. Anemia directly contributed to elevated CBF and hypertension affected CBF through anemia. Unaffected CVR of young patients probably indicated that they could maintain basic functions of cerebral circulation under multiple risk factors.

Altered cerebral autoregulation is common in uremic hypertension^1^. By local vasomotor adjustments in cerebral vascular resistance (CVR), cerebral autoregulation mechanism keeps CBF relatively constant to ensure tight coupling between oxygen supply and brain oxygen demand in healthy subjects^2^. In end-stage renal disease (ESRD) patients, elevated cerebral blood flow (CBF) had been repeatedly reported^3^,^4^,^5^,^6^. Anemia-induced hypoxia caused low cerebral oxygen delivery (COD) which was thought to play a key role in cerebral hyperperfusion in ESRD patients^5^. To make up low oxygen supply, CBFs of ESRD patients were increased to maintain basic oxidative metabolisms^4^,^5^. Hypertension may also contribute to cerebral circulation disorders in ESRD patients. Coinciding with hypertension, low arterial wall distensibility and increased CVR have been reported in ESRD patients^7^,^8^. It seems that anemia and hypertension are coupled risk factors of cerebral hyperperfusion in ESRD patients. However, the relationships among them are still unclear.

The average age of patients with ESRD in China is much younger than United State and Japan^9^. About 90% ESRD patients undergoing dialysis received hemodialysis (HD)^9^. And hence, there is a large population of young ESRD adults undergoing HD. Young HD patients might afford a better hemodynamic tolerance than elder ones. However, their cerebral circulation disorders are often overlooked because of their age. In this study, we aim to expore the relationships among anemia, hypertension and cerebral hyperperfusion in young HD patients. Also, COD and CVR were quantified to identify oxygen supply and vascular reaction in these patients, respectively. The relationships between changes in cerebral circulation and their risk factors and the patterns of CBF, COD and CVR may improve our knowledge about impaired cerebral autoregulation in HD patients.

## Results

### Clinical and laboratory data

The clinical and laboratory data of the subjects in this study are shown in Table 1. The age and gender were not significantly different between HD patients and healthy controls (both P > 0.05). Our patients were suffered from serious hypertension and their systolic and diastolic blood pressures were significantly higher than those of healthy subjects (both P < 0.001). Anemia was commonly found in HD patients who had significantly lower hemoglobin compared with healthy subjects (P < 0.001).

### Changes in tCBF, CVR and COD

Table 2 illustrates the total CBF (tCBF), CVR and COD of HD and healthy groups. HD patients had significantly higher tCBF compared with healthy subjects (P < 0.001, Bonferroni corrected). Although increased tCBF can help deliver more oxygen to brain, HD patients had significantly lower total COD compared with healthy subjects (P < 0.001, Bonferroni corrected) because of their severe anemia. There was no difference in CVR between HD patients and healthy controls (P = 0.79; Bonferroni corrected).

### Correlation results

**Figure 1:**
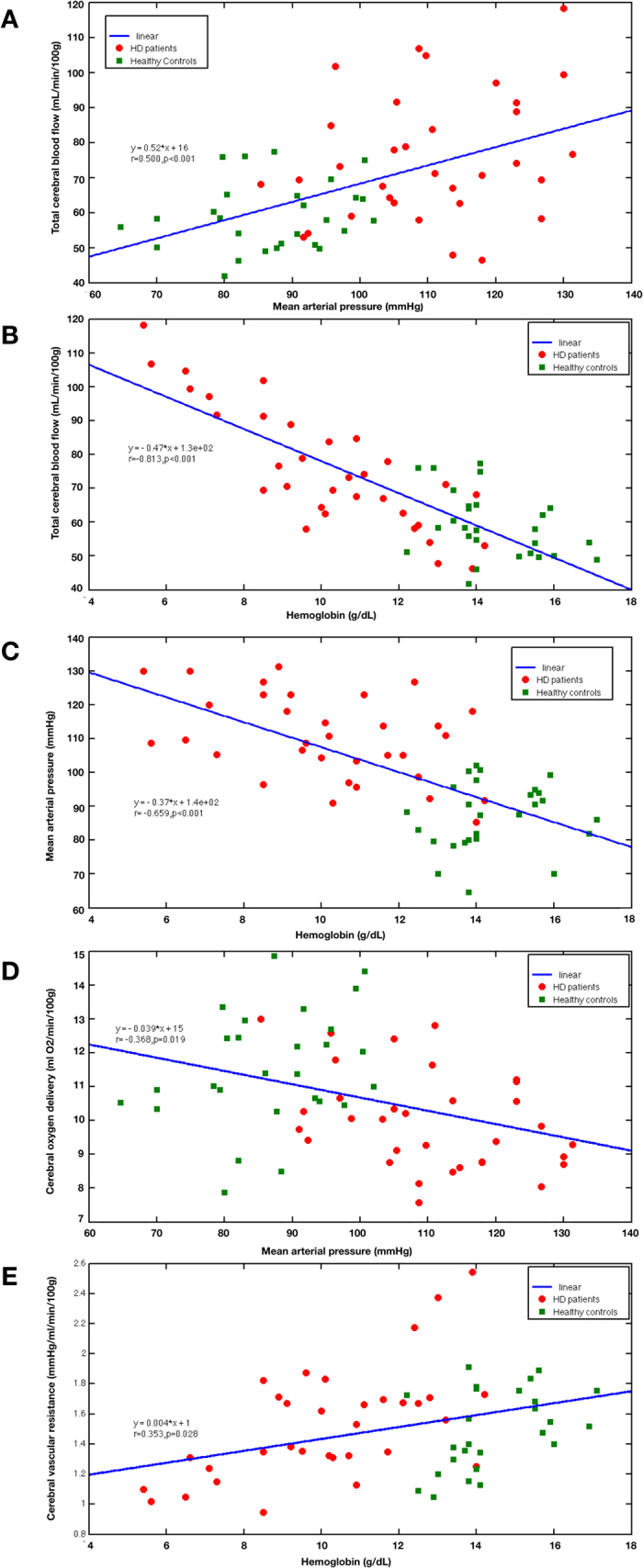
The Pearson cross correlations among cerebral brain flow, oxygen delivery, vascular resistance, hemoglobin and mean arterial pressure of young end-stage renal disease adults undergoing hemodialysis. (**A**) Cerebral blood flow and mean arterial pressure; (**B**) Cerebral blood flow and hemoglobin; (**C**) Mean arterial pressure and hemoglobin; (**D**) Cerebral oxygen delivery and mean arterial pressure; (**E**) Cerebral vascular resistance and hemoglobin. Note: Correlation p values were Bonferroni corrected.

**Figure 2:**
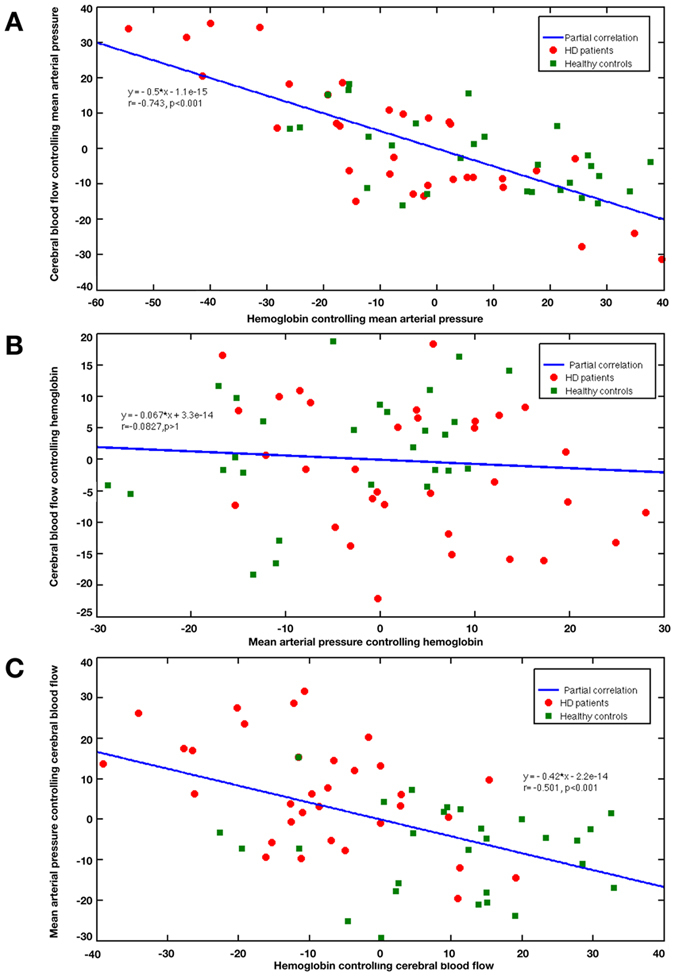
The partial correlations among cerebral blood flow, hemoglobin and mean arterial pressure. (**A**) Cerebral blood flow and hemoglobin controlling mean arterial pressure; (**B**) Cerebral blood flow and mean arterial pressure controlling hemoglobin; (**C**) Hemoglobin and mean arterial pressure controlling cerebral blood flow. Note: Correlation p values were Bonferroni corrected.

There were strong relationships between tCBF, mean arterial pressure (MAP) and hemoglobin (Pearson correlation; all P < 0.001; Bonferroni corrected; Fig. 1A–C). Decreased COD was correlated with elevated MAP (Pearson correction; r = **−**0.368, P = 0.019; Bonferroni corrected; Fig. 1D). CVR was positively correlated with hemoglobin (Pearson correlation; r = 0.353, P = 0.028; Bonferroni corrected; Fig. 1E). Factoring out the contribution of MAP, CBF was negatively correlated with hemoglobin (Partial correlation; r = **−**0.743, P < 0.001; Bonferroni corrected; Fig. 2A). However, CBF was not related to MAP after controlling hemoglobin (Partial correlation; r = **−**0.0827, P > 1; Bonferroni corrected; Fig. 2B). Significant partial correlation was still observed between MAP and hemoglobin while controlling CBF (Partial correlation; r = **−**0.501, P < 0.001; Bonferroni corrected; Fig. 2C).

Table 3 shows the blood flows, velocities and cross-sectional areas of four feeding arterials of HD and control groups. The blood flows of bilateral internal carotid arteries (ICAs) and right vertebral artery (VA) significantly increased in HD patients compared with controls (All P < 0.01, FDR corrected). The average flow velocity of HD patients significantly increased in bilateral VAs (Both P < 0.01, FDR corrected), but not in bilateral ICAs (Both P > 0.6, FDR corrected). The maximum, minimum and average cross-sectional areas of bilateral ICAs were significantly larger in HD patients compared with controls (All P < 0.05, FDR corrected), whereas the maximum, minimum and average cross-sectional areas of bilateral VAs were not different between two groups (All P > 0.4, FDR corrected). The total cross-sectional area of the four feeding arterials of HD patients was significantly greater than that of healthy subjects (P = 0.009, FDR corrected).

Pearson cross correlations showed that both hemoglobin and MAP were related with blood flows, velocities and cross-sectional areas of feeding arterials (Table 4). Factoring out the contribution of MAP, hemoglobin was still negatively correlated with blood flows and average velocities of four arterials (Partial correlations; all P < 0.05; FDR corrected; Table 4), with peak velocity of bilateral VAs (Partial correlations; all P < 0.01; FDR corrected; Table 4), and with the cross-sectional area of left ICA. But, MAP was not correlated with any of hemodynamic measurements of four feeding arterials after controlling the contribution of hemoglobin (Partial correlations; all P > 0.05; FDR corrected; Table 4).

The serum calcium levels of our patients (2.21 ± 0.16 mmol/L) were nearly normal (normal range: 2.02–2.60 mmol/L), whereas their phosphorus levels (2.04 ± 0.47 mmol/L) were higher than normal level (normal range: 0.81–1.65 mmol/L). There were significant correlations in HD patients between calcium and tCBF or hemoglobin (Pearson correlations; r = **−**0.44, P = 0.01, and r = 0.45, P = 0.009, respectively; uncorrected). No correlation was found among patients between their MRI measurements and their hemodialysis duration, phosphorus, or calcium*phosphorus (Pearson correlations; all P > 0.05; uncorrected).

## Discussion

This study found that there were strong correlations among elevated MAP, decreased hemoglobin and elevated CBF in young ESRD HD adults. Hemoglobin level was negatively correlated with CBF, whereas MAP was not related to CBF after controlling hemoglobin. Moreover, we found decreased COD and unchanged CVR of HD group compared with controls. Increased CBFs can be attributed to dilated bilateral ICAs and increased flow velocity of bilateral VAs. The relationships among anemia, hypertension and CBF and the patterns of changes in CBF, COD and CVR provide us a better understanding about the abnormal autoregulation mechanism in young ESRD HD adults.

Elevated CBF was found in ESRD HD patients, which directly attributed to anemia rather than hypertension. Increased CBF in CKD patients has been repeatedly reported by multimodality imaging studies, such as ^133^Xe inhalation technique^3^, ^15^O positron emission tomography^4^,^5^, arterial spin labeling MRI^6^ and Doppler ultrasonography^10^. Our findings based on pcMRI were consistent with previous studies^3^,^4^,^5^,^6^,^10^, supporting that pcMRI technique could provide reliable results. Specially, the measured blood velocity and blood flow were not sensitive to T1 or T2 of the spins because pcMRI utilizes the phase of an image to encode the velocity of moving spins. Anemia in ESRD patients was mainly caused by the declined erythropoietin production^11^,^12^. Low hemoglobin tended to reduce both blood viscosity and oxygen supply. However, the change in viscosity per se did not result in the hypoxia-induced increase in CBF^13^. Thus, brain tissue hypoxia caused by anemia could be the most important factor of elevated CBF in ESRD patients^5^. Similar with anemia, hypertension is also common in ESRD patients. Coinciding with renal dysfunction, the renin–angiotensin–aldosterone-system can be activated, which has been implicated in pathogenesis of hypertension^14^. Although MAP was correlated with CBF, it was not related to CBF after controlling hemoglobin, supporting that hypertension was not a direct risk factor of elevated cerebral hyperperfusion. Thus, the correlation between MAP and CBF could be caused by the strong correlation between hemoglobin and CBF.

Low COD played an important role in cerebral vascular changes in HD patients. Low oxygen saturation^15^ and low oxygen supply^5^ of ESRD patients had been reported. In our study, COD was lower in HD patients because of their severe anemia. Increased CBF would deliver more arterial blood to the brain, however, such a compensational procedure was not sufficient to make up hypoxia in ESRD patients^4^. Hypoxia could reduce the amount of ATP which causes ATP-gated ion channels in smooth muscle cells to open and hyperpolarize^16^. Hyperpolarization could reduce contractile ability, which could induce arteries to dilate. New *et al*.^1^ reported that vessel diameter was significantly greater in uremic Wistar-Kyoto rats compared with normotensive controls. In this study, we observed a significant increase of total cross-sectional arterial area and negative correlation between hemoglobin level and arterial area, indicating that anemia-induced hypoxia might cause cerebral vascular changes to deliver more blood to compensate the oxygen limitation. Also, we observed negative correlation between COD and MAP, supporting that HD patients with severe hypoxia might suffer from more serious hypertension.

Unchanged CVR of HD patients could be an outcome of anemia and vasodilation. Heyman *et al*. reported increased CVR in patients with uremia^8^. Our finding was not consistent with theirs, possibly because our patients were much younger and had more severe renal disease compared with their patients. CVR was reported to relate with degree of anemia^17^. We also observed such a positive correlation between CVR and hemoglobin in anemic patients. Low hemoglobin level might be associated with low blood viscosity^18^. Besides, we detected vasodilation in bilateral ICAs. According to the law of Poiseuille, unchanged CVR in HD patients can be explained by the decreased viscosity and increased vessel diameter. Young HD patients in our study had unchanged CVRs and free of severe cerebrovascular disease, supporting that young HD adults might maintain basic functional of cerebral circulation system under multiple risks of vascular disease.

The mechanisms of elevated blood flow were different between ICA and VA. Bilateral VAs increased flow velocities and kept arterial areas unchanged to support elevated blood flow. Our findings about elevated velocities of VAs were consistent with one previous study based on Doppler sonography^10^. However, the cross-sectional areas of VAs were not significantly changed in our study, which was not consistent with theirs^10^. Our patients were much younger and had much higher blood pressure than theirs. It seemed that the cross-sectional areas of VAs were restricted by the cervical vertebrae or by the transverse foramen, which can limit the further dilatation of VAs. Elevated velocities of bilateral VAs were associated with increased MAP, indicating that blood flows of VAs were increased through elevated velocities. Being different from VAs, bilateral ICAs increased blood flow through arterial dilatation rather than increased velocities. Both maximal and minimal cross-sectional areas of bilateral ICAs were significantly increased, leading to a significantly increased total cross-sectional area of feeding arterials.

Young ESRD HD adults were of high risks of cerebrovascular disease. Abnormalities in calcium and phosphorus metabolism played an important factor of causing vascular calcification^19^. To improve bone metabolism as well as to prevent progression of vascular calcification, the calcium containing phosphate binders were generally provided to HD patients in our hospital. Coinciding with high serum phosphorus concentrations and a high calcium–phosphorus ion product in serum, vascular calcification was common and progressive in young adults with ESRD undergoing dialysis^20^. Our patients were of abnormal high phosphorus and normal calcium levels, indicating that they would have much higher risk of vascular calcification. The positive relation between serum calcium level and CBF were found in our patients. The dialysate calcium concentration for HD patients is generally adjusted to optimize calcium and phosphate balance. The coupling between calcium and CBF could be caused by the adjustment dialysate calcium concentration. Further study is needed to find the mechanism of this coupling.

We acknowledge that there were several limitations in this study. First, only young ESRD HD adults were included in this study. Different dialysis modalities for ESRD patients could affect their cerebral circulation system in different ways. In future studies, patients treated by peritoneal dialysis, non-dialysis patients with ESRD as well as patients undergoing renal transplantation should be included to verify our findings. Second, individual intracranial pressure (ICP) was not available in this study. ICP was generally much lower than MAP and was ignored in the definition of CVR^17^. In our study, no medical record showed that young ESRD HD patients had significantly increased ICP. All our subjects were free of encephalopathy and brain injury. And hence, the ignored ICP term in the CVR definition would not significantly affect the conclusion about CVR. Third, the arterial oxygen saturation was assumed to be 98% in our study. The arterial oxygen saturation was not affected in HD patients^4^. Thus, our assumption about the arterial oxygen saturation would not significantly affect our findings.

## Materials and Methods

### Subjects

This prospective study was approved by the Jinling hospital Medical Research Ethics Committee, and all experimental protocols were performed in accordance with relevant guidelines and regulations. All participants gave written consent forms before MRI scans. Thirty-three young ESRD adults undergoing HD (23 males, 10 females, age from 18 to 35 years, mean age 26.6 ± 5.4 years) were recruited in this study. Patient including criteria were: young adults ageing from 18 to 35 years old, no clinical symptoms of encephalopathy, and no any MRI contraindications. Twenty-seven age- and gender- matched healthy subjects (18 males, 9 females, age from 21 to 35 years, mean age 25.0 ± 2.6 years) were recruited from local community. All healthy subjects had no diseases affecting brain functions. No drug abuse history was reported. Abdominal ultrasound scans revealed no abnormal findings for all healthy subjects.

Brachial artery blood pressure of each subject was measured by an automatic sphygmomanometer (Omron HEM 1000, Omron electronics LLC, Japan). The measurements were performed at rest before MRI examination and the average value of three consecutive measurements over ten minutes was computed. All subjects took routine blood test right after MRI examination. HD patients took additional blood biochemistry tests, including serum creatinine level, calcium and phosphorus concentrations.

### Imaging acquisition

All the MRI examinations were performed on a clinical 3T whole-body MR scanner (Siemens TIM Trio, Siemens Medical Solutions, Erlangen, Germany). High resolution T1-weighted images were acquired by the 3D MPRAGE sequence for the localization of pcMRI and for individual CBF quantification. The cardiac-triggered pcMRI scans were positioned perpendicular to the feeding arteries. The imaging parameters of pcMRI were as follows: field of view = 200 * 200 * 5 mm^3^, based resolution = 256, single slice, TR = 38.15 ms, TE = 3.47 ms, flip angle = 25 deg, segments = 3, velocity encoding = 120 cm/s, bandwidth = 543 Hz/Px. All subjects were instructed to stay still, and not think about anything with eyes closed during MRI scans.

### Quantification of tCBF, CVR and COD

**Figure 3:**
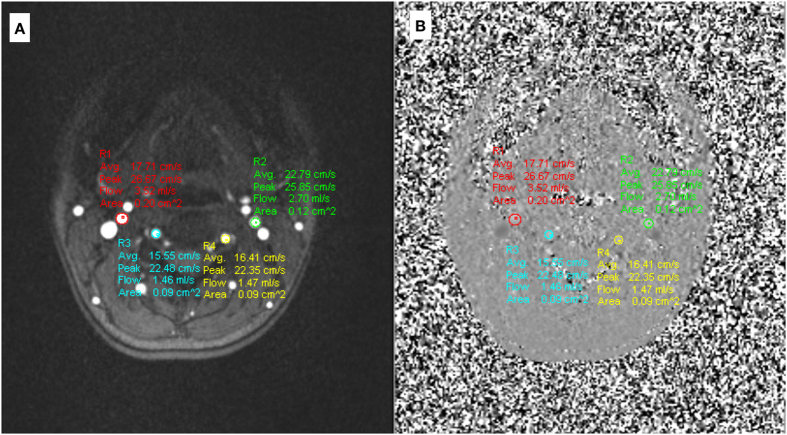
The magnitude and phase images of phase contrast MRI. (**A**) The magnitude image; (**B**) The phase image. Notes: Four regions of interests were drawn on the magnitude image. R1/R2 = right/left internal carotid artery; R3/R4 = right/left vertebral artery. The circles in R1-R4 illustrate the voxels with maximal velocities.

The tCBF is measured by the pcMRI technique applied at the four feeding arteries (i.e. bilateral ICAs and bilateral VAs at the bottom of the brain. In this paper, the blood flow (BF, in unit ml/s) of each artery is quantified on the phase image of pcMRI images by the Siemens’s product software Argus. Firstly, both magnitude and phase series are loaded into Argus Viewer. Secondly, the contour of each artery is drawn on the first magnitude image (Fig. 3A), which will be also created on the phase image (Fig. 3B). Thirdly, the four contours of the feeding arterials are auto adjusted and then propagated within slice. The first image is discarded to reduce anthropogenic influence. Finally, the results about velocities, flows and cross-sectional areas of arteries are reported in the summary table of Argus.

The tCBF (in unit 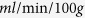) is calculated by summing the blood flows of the four feeding arteries via:


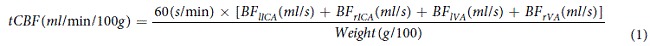


where 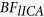, 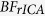, 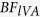, and 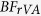 are the blood flow velocities of left ICA, right ICA, left VA and right VA, respectively. 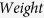 in Eq. (1) represents the total weight of a given brain and is used to adjust tCBF. Suppose that the density of the brain tissue is 1.06 g/ml, the brain weight can be calibrated by


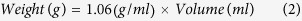


where 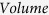 is the total volume of gray and white matter which are obtained by partitioning the 3D T1 weighted image based on SPM8 (Statistical Parametric Mapping, http://www.fil.ion.ucl.ac.uk/spm/).

CVR can be calculated as follows:


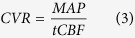


where 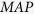 represents mean arterial pressure. 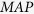 is calculated by


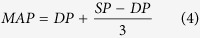


where 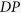 and 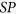 are diastolic and systolic pressure, respectively.

Generally, the amount contributed by dissolved oxygen in plasma is very small. In this paper, the oxygen dissolved in plasma is omitted. Suppose that 1.0 g of hemoglobin binds 1.39 ml oxygen^21^, COD can be computed by:


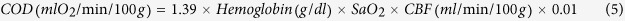


where 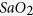 represents the arterial oxygen saturation. Because 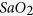 is known to be relatively stable, it is assumed to be 98%.

### Statistical analysis

Statistical analyses were performed by the software IBM SPSS statistics (version 22). The tCBF, COD, and CVR were analyzed with an ANCOVA model to detect hemodynamic differences between HD patients and controls. Age and gender were included in ANCOVA as nuisance covariates. Correlations between aforementioned global measurements and MAP or hemoglobin were computed based on Pearson cross correlation. Partial correlation was performed among tCBF, MAP and hemoglobin to control the interactions among them. To control the multiple competitions among tCBF, COD, and CVR, a Bonferroni-corrected P less than 0.05 was considered as significant.

One-way ANCOVA was performed to compare pcMRI measurements between two groups. Correlations between pcMRI measurements and MAP or hemoglobin were calculated based on Pearson cross correlation and partial correlation. The false discovery rate (FDR) was applied to correct the multiple comparisons among multiple pcMRI measurements. An FDR-corrected P value less than 0.05 was considered as significant.

Correlations were also performed within HD patients to explore the relationships between the MRI measurements and HD duration, calcium, phosphorus, or the product of calcium and phosphorus. The significance level was set at a p value less than 0.05 without correction.

## Additional Information

**How to cite this article**: Zheng, G. *et al*. Anemia rather than hypertension contributes to cerebral hyperperfusion in young adults undergoing hemodialysis: A phase contrast MRI study. *Sci. Rep.* **6**, 22346; doi: 10.1038/srep22346 (2016).

## Figures and Tables

**Table 1:** Clinical and laboratory characteristics of the healthy subjects and hemodialysis patients.

	Healthy Controls	Hemodialysis patients	P value
Demographic			
Age (Y)	25.5 ± 2.7	26.6 ± 5.4	0.36^**|**^
Gender (M/F)	18/9	23/10	0.51^**!**^
Blood pressure, mmHg			
Systolic	113.1 ± 13.8	145.9 ± 17.9	**<0.001**^**@**^
Diastolic	74.0 ± 8.9	92.4 ± 11.2	**<0.001**^**@**^
Mean arterial	87.0 ± 9.9	110.2 ± 12.6	**<0.001**^**@**^
Blood tests			
Hemoglobin, g/dl	14.4 ± 1.3	10.2 ± 2.4	**<0.001**^**@**^
Creatinine, mg/dl	—	11.7 ± 3.2	—
Calcium, mmol/L	—	2.21 ± 0.16^a^	—
Phosphorus, mmol/L	—	2.04 ± 0.47^b^	—
Calcium * Phosphorus	—	4.50 ± 1.10	—
Dialysis vintage (months)	—	10.8 ± 16.1	—
Etiology	CGN n = 19; IgAN n = 6; SLE and CGN n = 1; CGN and IgAN n = 1; Ureterostenosis n = 1; Fabry’s disease n = 1; Diabetes n = 1; Unknown n = 3.		

Values are mean ± SD or number of patients;

^|^stands for the result of the two sample t test;

^**!**^stands for the results of the Chi-square test;

^@^stands for the result of the one way ANCOVA with age and gender as covariances;

^a^The normal range of serum calcium is 2.02–2.60 mmol/L in our hospital;

^b^The normal range of serum phosphorus is 0.81–1.65 mmol/L in our hospital;

CGN = chronic glomerulonephritis, IgAN = immunoglobulin A nephropathy, SLE = systemic lupus erythematosus.

**Table 2:** One-way ANCOVA comparison of total cerebral blood flow, cerebral oxygen delivery, and cerebral vascular resistance between young adults with ESRD undergoing hemodialysis and healthy controls.

Variables	tCBF 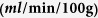	COD 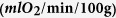	CVR 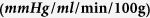
ESRD patients	**75.7** ± **18.1**	**10.0** ± **1.4**	1.53 ± 0.37
Healthy controls	**59.1** ± **9.7**	**11.5** ± **1.7**	1.51 ± 0.26
ANCOVA F values	**19.2**	**14.6**	0.07
ANCOVA P values	**<0.001**	**<0.001**	0.79

Note: ANCOVA P values were Bonferroni corrected.

ESRD = end-stage renal disese; tCBF = total cerebral blood flow; COD = cerebral oxygen delivery; CVR = cerebral vascular resistance.

**Table 3:** Comparisons of cerebral blood flows, velocities and areas of four feeding arterials between hemodialysis patients and healthy subjects.

Variables	Hemodialysis patients	Healthy subjects	ANCOVA F values	ANCOVA P values
Average flow over range, ml/s				
Right ICA	**5.80** ± **1.64**	**4.51** ± **0.95**	**12.78**	**0.004**
Left ICA	**5.22** ± **1.60**	**4.03** ± **0.64**	**13.63**	**0.003**
Right VA	**1.87** ± **0.83**	**1.27** ± **0.70**	**8.96**	**0.009**
Left VA	2.26 ± 0.83	1.97 ± 0.65	2.31	0.214
Peak velocity, cm/s				
Right ICA	42.1 ± 9.8	46.3 ± 9.1	3.11	0.139
Left ICA	41.3 ± 11.0	43.9 ± 8.6	1.10	0.440
Right VA	**34.9** ± **7.3**	**30.6** ± **6.2**	**6.12**	**0.028**
Left VA	37.8 ± 9.7	36.0 ± 4.7	0.89	0.462
Average velocity, cm/s				
Right ICA	22.4 ± 6.5	22.4 ± 5.3	0.00	0.987
Left ICA	23.1 ± 6.6	22.2 ± 5.0	0.38	0.641
Right VA	**17.0** ± **3.7**	**13.4** ± **3.0**	**18.07**	**0.001**
Left VA	**19.0** ± **4.4**	**15.8** ± **2.7**	**11.73**	**0.004**
Average cross-sectional area, cm^2^				
Right ICA	**0.273** ± **0.087**	**0.213** ± **0.063**	**9.53**	**0.008**
Left ICA	**0.238** ± **0.085**	**0.189** ± **0.048**	**7.23**	**0.020**
Right VA	0.107 ± 0.037	0.103 ± 0.028	0.19	0.829
Left VA	0.117 ± 0.028	0.125 ± 0.039	0.89	0.479
Minimal cross-sectional area, cm^2^				
Right ICA	**0.241** ± **0.079**	**0.189** ± **0.045**	**9.39**	**0.008**
Left ICA	**0.211** ± **0.078**	**0.169** ± **0.037**	**6.87**	**0.039**
Right VA	0.102 ± 0.037	0.100 ± 0.027	0.07	0.829
Left VA	0.113 ± 0.027	0.120 ± 0.039	0.76	0.479
Maximal cross-sectional area, cm^2^				
Right ICA	**0.304** ± **0.098**	**0.234** ± **0.075**	**9.72**	**0.008**
Left ICA	**0.262** ± **0.095**	**0.213** ± **0.063**	**5.37**	**0.039**
Right VA	0.112 ± 0.036	0.114 ± 0.033	0.07	0.829
Left VA	0.122 ± 0.031	0.131 ± 0.041	1.08	0.440
Total cross-sectional area, cm^2^	**0.735** ± **0.149**	**0.631** ± **0.128**	**8.60**	**0.009**

Note: ANCOVA P values were FDR corrected.

ICA = internal carotid artery; VA = vertebral artery

**Table 4:** Relationships between hemodynamic measurements of four feeding arterials, mean arterial pressure, or hemoglobin level.

Variables	MAP		Hb	
	Pearson correlation	Partial correlation controlling Hb	Pearson correlation	Partial correlation controlling MAP
Right ICA	0.440**	0.042	−0.630***	−0.503***
Left ICA	0.432**	**−**0.149	−0.764***	−0.707***
Right VA	0.338*	0.038	−0.474***	−0.356*
Left VA	0.209	**−**0.159	−0.476***	−0.460**
Peak velocity				
Right ICA	**−**0.100	**−**0.243	**−**0.124	**−**0.253
Left ICA	**−**0.096	**−**0.258	**−**0.146	**−**0.279
Right VA	0.319*	**−**0.038	−0.521***	−0.436**
Left VA	0.209	**−**0.128	−0.447**	−0.421**
Average velocity				
Right ICA	0.115	**−**0.118	−0.303*	−0.305*
Left ICA	0.123	**−**0.114	−0.310*	−0.307*
Right VA	0.496***	0.079	−0.688***	−0.553***
Left VA	0.499***	0.143	−0.630***	−0.462**
Average cross**−**sectional area				
Right ICA	0.315*	0.180	−0.281*	**−**0.103
Left ICA	0.290	0.013	−0.426**	−0.327*
Right VA	0.015	**−**0.027	**−**0.054	**−**0.059
Left VA	**−**0.147	**−**0.263	**−**0.076	**−**0.232
Minimal cross**−**sectional area				
Right ICA	0.302	0.178	**−**0.263	**−**0.089
Left ICA	0.264	**−**0.005	−0.405**	−0.319*
Right VA	**−**0.017	**−**0.051	**−**0.033	**−**0.058
Left VA	**−**0.132	**−**0.251	**−**0.084	**−**0.230
Maximal cross-sectional area				
Right ICA	0.334*	0.182	−0.309*	**−**0.126
Left ICA	0.271	0.007	−0.405**	−0.312*
Right VA	**−**0.060	**−**0.061	0.021	**−**0.024
Left VA	**−**0.114	**−**0.241	**−**0.100	**−**0.235
Total cross-sectional area, cm^2^	0.289	0.039	−0.398**	−0.288*

Note: The data represent the coefficients of Pearson correlations or partial correlations.

*stands for FDR-corrected P < 0.05; **stands for FDR-corrected P < 0.01; ***stands for FDR-corrected P < 0.001. ICA = internal carotid artery; VA = vertebral artery; MAP = mean arterial pressure; Hb = hemoglobin.
